# Effects of Orbit and Pointing Geometry of a Spaceborne Formation for Monostatic-Bistatic Radargrammetry on Terrain Elevation Measurement Accuracy

**DOI:** 10.3390/s90100175

**Published:** 2009-01-08

**Authors:** Alfredo Renga, Antonio Moccia

**Affiliations:** Department of Aerospace Engineering, University of Naples Federico II, 80 Piazzale Tecchio, 80125, Naples, Italy; E-Mail: antonio.moccia@unina.it

**Keywords:** Spaceborne Monostatic-Bistatic Synthetic Aperture Radar, Single-Pass Stereo-Radargrammetry, Space Mission Design, Terrain Elevation Measurement Accuracy

## Abstract

During the last decade a methodology for the reconstruction of surface relief by Synthetic Aperture Radar (SAR) measurements – SAR interferometry – has become a standard. Different techniques developed before, such as stereo-radargrammetry, have been experienced from space only in very limiting geometries and time series, and, hence, branded as less accurate. However, novel formation flying configurations achievable by modern spacecraft allow fulfillment of SAR missions able to produce pairs of monostatic-bistatic images gathered simultaneously, with programmed looking angles. Hence it is possible to achieve large antenna separations, adequate for exploiting to the utmost the stereoscopic effect, and to make negligible time decorrelation, a strong liming factor for repeat-pass stereo-radargrammetric techniques. This paper reports on design of a monostatic-bistatic mission, in terms of orbit and pointing geometry, and taking into account present generation SAR and technology for accurate relative navigation. Performances of different methods for monostatic-bistatic stereo-radargrammetry are then evaluated, showing the possibility to determine the local surface relief with a metric accuracy over a wide range of Earth latitudes.

## 1. Introduction

Bistatic radar is today a research field of great and renewed interest. Recent studies have revealed the potential of spaceborne bistatic radar for large-scale Earth observation by using transmitting and receiving antennae flying in formation for integrated operation [1-5]. Moreover, nowadays a great demand exists for Digital Elevation Models (DEM) on wide areas for an ample range of commercial and scientific applications [6]. The most mature method for wide area DEM generation by means of microwave space remote sensing is SAR Interferometry (InSAR) [7]. InSAR coherently combines the signals from two SAR antennae to calculate the interferometric phase difference in each image point. The phase difference directly depends on the local relief. However, the phase interferogram must be unwrapped to resolve the modulo 2 ambiguity before estimating topography. Different approaches exist for phase unwrapping [7], usually a time-consuming and difficult task that cannot be avoided. In forested or mountain slope areas phase coherence can be very low, and an uncorrected solution of the phase unwrapping problem might be derived, often leading to diverging errors in interferometric DEM generation [8].

InSAR is not the only technique for DEM generation from SAR data [9]: a previously existing technique is Stereo-Radargrammetry [10]. Both InSAR and Stereo-Radargrammetry are based on the notion of detecting relative displacements between two images, except that InSAR uses phase domain data and, operating on a wavelength scale, is intrinsically more accurate, but also less robust if phase decorrelation is strong [11, 12], whereas Stereo-Radargrammetry exploits only range/Doppler and amplitude measurements. Stereo-Radargrammetry is able to provide good results only for large stereo intersection angles [13], for this reason it was applied only on repeat-track spaceborne SAR data [8, 13-16]. However, according to the present trend in bistatic spaceborne SAR missions [1, 2], the possibility of forming a very large (from tens to hundreds of kilometers) controlled separation between the antennae (baseline) by means of two satellites operating simultaneously and flying in formation is concrete.

In a previous work [17] the authors analyzed stereo-radargrammetric methods applied to spaceborne monostatic-bistatic SAR with reference to an observation geometry based on parallel tracks, i.e. monostatic and bistatic antennae flying in formation along parallel trajectories at the same height, with identical velocities and with no squint angles. These hypotheses represent a simplification of actual spaceborne scenarios, since it is not possible to achieve perfectly parallel orbits, so differences in antenna altitudes, velocities and pointing angles between monostatic and bistatic sensor exist. The main goal of this paper is to investigate performance of a spaceborne monostatic-bistatic SAR mission accounting for actual and time-varying orbital configuration. To this end, first of all key orbital design issues for spaceborne monostatic-bistatic acquisitions able to assure single-pass stereo-radargrammetric coverage are pointed out. After introducing models adequate to derive terrain height measurements from stereo radargrammetric pairs, a performance evaluation is carried out considering the Italian COSMO-SkyMed mission [18, 19] as reference for the monostatic satellite and a bistatic receiving-only sensor flying in formation with it [2, 20]. Finally, simulation results are presented, showing quantitatively the height measurement accuracy that can be achieved and its time history along the orbit.

## 2. Monostatic-Bistatic Geometry for Stereo-Radargrammetric Reconstruction

Relief reconstruction by spaceborne Stereo-Radargrammetry is based on the detection of target relative displacements between two SAR images and on the equations relating the positions and heights of the viewed targets [9]. Different acquisition geometries are exploitable if two monostatic images are used [13]: same and opposite sides, steep and shallow look angles. From a geometric point of view larger stereo-intersection angles (i.e. very large baselines and opposite-side stereo pairs) provide the best results [9]. Actually, when dealing with real-world data, large radiometric discrepancies are generated in opposite-side geometry thus limiting previous advantages [21], especially as terrain slope [14] and time decorrelation effects due to non-simultaneity between the images [11] increase. Therefore, provided that a minimum intersection angle is guaranteed in repeat-pass same-side geometry (8° has been suggested in [22]), it is possible to state that there is not a significant correlation between DEM accuracy and acquisition geometry.

In a spaceborne monostatic-bistatic scenario further considerations are needed:

- the bistatic image quality in terms of resolutions, ambiguities, signal-to-noise-ratio (SNR) is greatly affected by the monostatic-bistatic configurations, even before stereo reconstruction, therefore the effect of the acquisition geometry on bistatic image parameters must be accounted for when selecting the optimum configuration for radargrammetric DEM generation;
- when a monostatic image and a bistatic one are adopted to form a stereoscopic pair, new relations are needed to define target height as a function of the peculiar parameters of monostatic-bistatic surveying geometry [23]. Namely, the models of classical stereo-radargrammetry can be specialized to the monostatic/bistatic configuration, but also new models can be developed [17, 24].

### 2.1. Monostatic-Bistatic Observation Strategies

**Figure 1.**
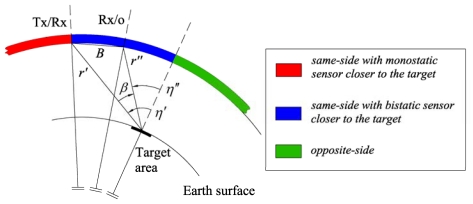
Observation strategies for monostatic-bistatic acquisition. The transmitting-receiving (Tx/Rx) and the receiving-only (Rx/o) antennae are supposed to operate at the same altitude in parallel trajectories (not to scale for clarity).

Geometric parameters characterizing the bistatic geometry are the baseline, *B*, that is the inter-antennae distance, and the bistatic angle, *β*, that is the transmitter-target-receiver angle [25]. It is worth noting that the bistatic angle plays the same role in monostatic-bistatic radargrammetry as the stereo-intersection angle in monostatic repeat-pass one. A general analysis of bistatic SAR resolution is reported in [26, 27], based on the gradient method and on the derivation of bistatic ambiguity function; literature results will be used in the next sections, whereas simplified expressions, based on [23], will be introduced here in order to assess basic rules for mission design. Assuming two space platforms operating at the same altitude in parallel trajectories, the bistatic ground range, Δ*r_g_″*, and azimuth, Δ*x*′ resolution can be related to the monostatic ones (Δ*r_g_′* and Δ*x′*, respectively) [23, 25]:

(1) $$\Delta {r^{\prime \prime }}_{g}=\frac{2\Delta {r^{\prime }}_{g}\sin\eta^{\prime }}{\sin\eta^{\prime }+\sin\eta^{\prime \prime }}$$

(2) $$\Delta x^{\prime \prime }=\frac{2r^{\prime \prime }}{r^{\prime }+r^{\prime \prime }}\Delta x^{\prime }$$

where *η′* and *η″* are monostatic and bistatic incidence angles; *r′* and *r″* are monostatic and bistatic slant ranges. Note that *η″* must be considered negative in opposite-side geometry (see Figure 1).

Theoretically, under the considered hypotheses, three different observation strategies can be realized (see Figure 1, where it is assumed constant the position of the monostatic SAR, and the different baselines are achieved thanks to adequate positioning of the bistatic receiving-only antenna):

- same-side with monostatic sensor closer to the target;
- same-side with bistatic satellite closer to the target;
- opposite-side.

**Figure 2.**
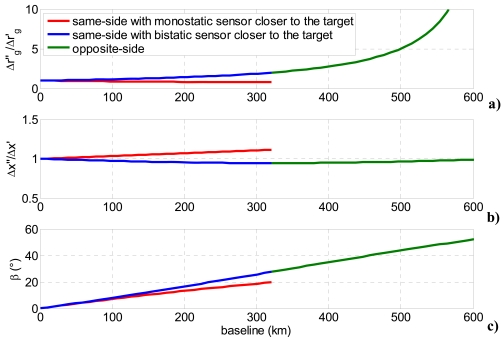
(a) Bistatic-to-monostatic ground range resolution ratio, (b) bistatic-to-monostatic azimuth resolution ratio, (c) bistatic angle, as a function of baseline (platforms operating at 620 km altitude in parallel trajectories).

Figure 2 shows the bistatic-to-monostatic ground range and azimuth resolution ratios with varying bistatic angle and observation strategy, considering a formation operating at 620 km altitude with constant 25 degrees monostatic off-nadir angle. Opposite-side geometry degrades bistatic ground range resolution considerably, whereas small variations of azimuth resolution can be estimated even if the worst values are realized for same-side configurations with monostatic sensor closer to the target.

**Figure 3.**
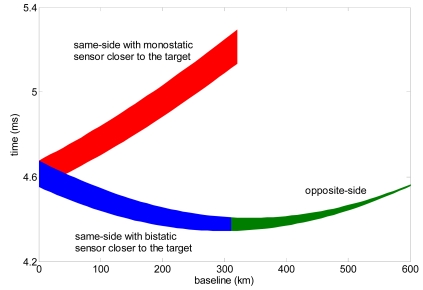
Bistatic time interval required to receive the same monostatic swath width (40 km) for different observation strategies as a function of the baseline (platforms operating at 620 km altitude in parallel trajectories).

Bistatic slant range ambiguities depend on system timing and monostatic/bistatic antenna patterns and observation geometries [25, 28]. An immediate way to evaluate the ambiguity level is to consider the time interval required for the bistatic antenna to receive the monostatic swath width in elevation, which is the time interval between reception of far range and near range echoes in bistatic geometry. Figure 3 shows the time intervals required for bistatic swath acquisition considering a footprint in elevation of 40 km. As expected, increasing baseline in opposite-side observation geometry makes very difficult to separate the backscattered signals from the different regions of the illuminated area. This condition is even worse when dealing with surface relief variations. Indeed, the actual terrain slope can produce locally the specular observation condition in which the bistatic range ambiguity is total [25].

Finally, assuming a Lambertian model both for monostatic and bistatic backscattering coefficients [3] the ratio between bistatic and monostatic SNR can be evaluated as in [31]:

(3) $$\frac{{\text{SNR}}^{\prime \prime }}{{\text{SNR}}^{\prime }}≅4\frac{{r^{\prime }}^{2}}{r^{\prime \prime }\left(r^{\prime }+r^{\prime \prime }\right)}\frac{\cos\eta^{\prime \prime }}{\cos\eta^{\prime }}\frac{\sin\eta^{\prime }}{\sin\eta^{\prime }+\sin\eta^{\prime \prime }}$$

**Figure 4.**
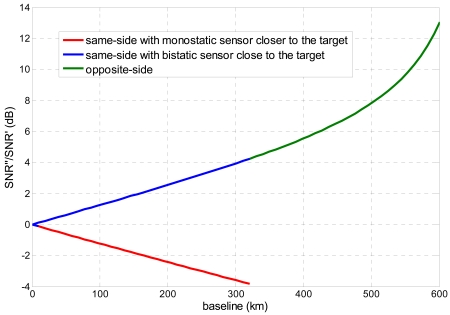
Ratio between bistatic and monostatic SNR for different observation strategies as a function of the baseline (platforms operating at 620 km altitude in parallel trajectories).

As shown in Figure 4, a regular trend in SNR ratio is derived for the same-side geometry, as the bistatic sensor approaches the target. The opposite-side geometry, on the contrary, exhibits a significant increase of bistatic SNR, however this is just a geometrical effect consequent to the worsening of the bistatic ground range resolution and therefore it can not be exploited. Of course the possibility to achieve high-quality relief reconstruction by stereo-radargrammetry relies on the availability of medium-to-high resolution bistatic SAR images.

### 2.2. Methods for Monostatic-Bistatic DEM Generation

There is not a unique strategy for radargrammetric relief reconstruction. In [17] five different methods are presented, considering simplified assumptions on monostatic-bistatic geometry. The first approach is derived from optical stereo photogrammetry and is based on the definition of the parallax difference between the two observations. A stereo parallax is created when an object is viewed from two different positions. Height computation by means of the parallax method is based on relative measurements, i.e. a datum must be defined and heights are computed with respect to datum level. Hence, the absolute parallax is defined with reference to the target relief displacement in the radar image, whereas parallax differences are obtained with respect to datum, which allows definition of a reference parallax that must be calculated to derive target relative height [23]. Finally, accurate knowledge of the height of a limited number of ground control points is needed to evaluate topography [29, 30].

The parallax method strongly relies on image quality and on capability to extract information from images, therefore the parallax method is the most sensitive to angle decorrelation and SNR degradation [17]. However, the intrinsic nature of radar, and of SAR too, allows direct access to slant range and Doppler measurements. On this basis the parallax difference can be expressed as a function of SAR measurements and there is no use in considering it as an independent parameter [17]. This statement is of fundamental importance to pass from optical derived approaches to true stereo radar methods, such as the rigorous stereo SAR problem [9] modified for monostatic/bistatic tandem acquisitions [31], or the hybrid approaches based both on the parallax and on the classic SAR range-azimuth measurements [24]. The first step of all these methods is the image matching: that is, relating a pixel of the bistatic image to the corresponding one in the monostatic image; in this way it is possible to associate both monostatic and bistatic SAR measurements (slant range, Doppler centroid frequency, ground range, azimuth…) of each observed target. For the sake of completeness it important to remark that the proposed monostatic-bistatic system is, no matter, narrow band SAR, and therefore speckle noise will be generated in the images. Robust pattern recognition algorithms shall be used to guarantee the sub-pixel co-registration [13] that is fundamental for accurate DEM generation.

A comprehensive analysis of methods for monostatic-bistatic DEM generation is available in [17]. In the following subsections the main relations for relief reconstruction are recalled, thus establishing the ground for performing a preliminary mission analysis and for producing an error budget of height estimation accuracy.

#### 2.2.1. Projection of Bistatic Parameters

**Figure 5.**
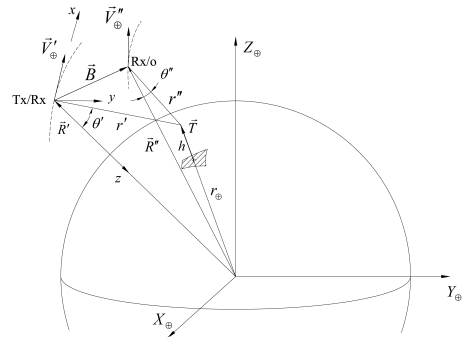
Three-dimensional viewing geometry of the monostatic-bistatic stereo-radargrammetric survey (not to scale for clarity).

The projection of bistatic parameters, such as baseline $\vec{B}$, slant range $\vec{r}\prime \prime$, sensor position $\vec{R}\prime \prime$, onto the range-elevation plane of the monostatic antenna allows three-dimensional monostatic-bistatic geometry to be accounted [32], and generates an explicit formulation for height determination [23]. The projection can be carried out considering the unit vector normal to the first antenna range elevation plane (Figure 5):

(4) $$\vec{n}=\frac{\vec{R}\prime \times \left({\overset{\prime }{V^{\to }}}_{⊕}\times \vec{R}\prime \right)}{\left|\vec{R}\prime \times \left({\overset{\prime }{V^{\to }}}_{⊕}\times \vec{R}\prime \right)\right|}$$

and, by means of this vector, calculating the projected parameters as follows:

(5) $$B_{⊥}=\sqrt{B^{2}-{\left(\vec{B}\cdot \vec{n}\right)}^{2}}$$

(6) $${r^{\prime \prime }}_{⊥}=\sqrt{{r^{\prime \prime }}^{2}-{\left({\vec{r}}^{\prime \prime 2}\cdot \vec{n}\right)}^{2}}$$

(7) $${R^{\prime \prime }}_{⊥}=\sqrt{{\left|\vec{R}\prime \prime \right|}^{2}-{\left(\vec{R}\prime \prime \cdot \vec{n}\right)}^{2}}$$

where $\vec{R}\prime$ is the monostatic sensor position, ${{\vec{V}}^{\prime }}_{⊕}$ is the monostatic antenna velocity with respect to the Earth-centred-Earth-fixed (ECEF) reference frame. Finally, within the monostatic range-elevation plane, target altitude, *h*, can be derived as follows

(8) $$h=\sqrt{{R^{\prime }}^{2}+{r^{\prime }}^{2}-2R^{\prime }r^{\prime }\cos\theta^{\prime }}-r_{⊕}$$

where $r_{⊕}$ is the spherical Earth radius and *θ′* is the monostatic radar-target off-nadir angle, that can be expressed as

(9) $$\theta^{\prime }=\cos^{-1}\left(\frac{{R^{\prime }}^{2}+B_{⊥}^{2}-{R^{\prime \prime }}_{⊥}^{2}}{2R^{\prime }B_{⊥}}\right)-\cos^{-1}\left(\frac{{r^{\prime }}^{2}+B_{⊥}^{2}-{r^{\prime \prime }}_{⊥}^{2}}{2r^{\prime }B_{⊥}}\right)$$

For the sake of simplicity a spherical Earth is assumed, but the method can be applied to more general zero relief regular surfaces.

#### 2.2.2. Bistatic Rigorous SAR Stereo Problem

The equations defining the rigorous SAR stereo problem in bistatic geometry are [17]:

(10) $$r^{\prime }=\left|\vec{R^{\prime }}-\vec{T}\right|\;$$

(11) $$r^{\prime \prime }=\left|\vec{R^{\prime }}+\vec{B}-\vec{T}\right|\;$$

(12) $${f^{\prime }}_{DC}=2\frac{\left({{\vec{V}}^{\prime }}_{⊕}-{\vec{V}}_{T}\right)\cdot \left({\vec{R}}^{\prime }-\vec{T}\right)}{\lambda \left|{\vec{R}}^{\prime }-\vec{T}\right|}$$

(13) $${f^{\prime \prime }}_{DC}=\frac{1}{\lambda }\left[\frac{\left({{\vec{V}}^{\prime }}_{⊕}-{\vec{V}}_{T}\right)\cdot \left({\vec{R}}^{\prime }-\vec{T}\right)}{\left|{\vec{R}}^{\prime }-\vec{T}\right|}+\frac{\left({{\vec{V}}^{\prime }}_{⊕}+{\vec{V}}_{B⊕}-{\vec{V}}_{T}\right)\cdot \left(\vec{R^{\prime }}+\vec{B}-\vec{T}\right)}{\left|\vec{R^{\prime }}+\vec{B}-\vec{T}\right|}\right]$$

where $\vec{T}$ and ${\vec{V}}_{T}$ are the target position and velocity vector, respectively; ${\vec{V}}_{B⊕}$ is the ECEF baseline vector time derivative, *λ* is the radar wavelength, and *f′_DC_* and *f′_DC_* are the monostatic and bistatic Doppler centroid frequencies. Eqs. (10)-(11) define monostatic and bistatic range spheres, while Eqs. (12)-(13) individuates monostatic and bistatic Doppler cones. Differently from classic photogrammetry where triangulation equations are applied on corresponding targets identified in photographic images by means of the optical stereo reconstruction obtained thanks to stereocomparators, monostatic and bistatic slant ranges and Doppler centroid frequencies are derived for each image point of co-registered data, as in numerical photogrammetry. As for monostatic radargrammetry least square methods can be used to derive target positions [29, 31], and target height too, accounting for spherical zero relief surface and assuming a static scene ${\vec{V}}_{T}=0$

#### 2.2.3. Hybrid

A hybrid formulation for height determination has been introduced in [24], moving from the idea to derive the parallax vector from classic SAR ground range and azimuth coordinates. The core of the method is to evaluate where a target, characterized by non-zero height, is projected on two-dimensional monostatic and bistatic images. Relief reconstruction equation is:

(14) $$h=\frac{{\vec{u}}_{z}\cdot \vec{a}}{{\left|{\vec{u}}_{z}\right|}^{2}}$$

where the parallax vector, $\vec{a}$, is defined as the difference of ground range and azimuth coordinates between monostatic and bistatic images; ${\vec{u}}_{z}$ depends on position and velocity of monostatic and bistatic sensors with respect to the centre of the target area [24]. Eq. 14 allows computation of target height with respect to a control point of known height and therefore it assumes a reference plane tangent to an Earth-centred sphere with radius defined by the control point [17, 24].

Both theoretical and implementation differences among the methods exist, however in [17] it is showed that the overall height uncertainty reveals the same order of magnitude for each approach and metric accuracy can be achieved provided that bistatic angles are larger than 5-10°.

## 3. Mission Design

The orbital design of a spaceborne monostatic-bistatic mission must be developed in order to attain required baselines, to reduce maintenance operations and, thanks also to an adequate pointing strategy, to permit the bistatic sensor to observe the area illuminated by the monostatic one [32]. Moreover, the analysis synthetized in the previous section allows the following requirements to be defined for designing a formation flying missions devoted to radargrammetric DEM generation:

- parallel orbits, to take advantage of stereo effect;
- bistatic angle larger than 5-10°;
- same-side stereo configuration, to avoid bistatic slant range ambiguities;
- bistatic sensor closer to the target thus limiting too large off-nadir angles for the bistatic receiver and benefiting by stronger echoes.

In a pendulum formation the minimization of propellant mass is achieved by assuring that the orbital perturbations due to Earth oblateness have the same effects on both satellite orbits [32-34]. To this end monostatic and bistatic satellites must share the same semi-major axis, eccentricity, and inclination. Moreover, if the orbits have the same argument of perigee, velocity differences are minimized too, aiding in reducing too large relative along track displacements. Since pendulum formation establishes the maximum baseline over equatorial area, and, simultaneously, a minimum bistatic angle must be guaranteed for relief reconstruction, the differences in right ascension of ascending node (RAAN) and mean anomaly can be selected in order to maximize the area on which the acquisition with a large bistatic angle can be achieved. On this basis they are established so that the bistatic sensor is in the elevation plane of the monostatic one when passing over the equator; in [32] a mathematical model is presented to evaluate them. Assuming the Italian COSMO-SkyMed [18, 19] as a reference for the monostatic mission (see Table 1) the range of monostatic off-nadir angle is from 23.3° to 43.7° and the monostatic satellite performs a classic yaw steering manoeuvre to reduce aerodynamic drag and to attain zero monostatic Doppler centroid frequency. Moreover, considering the bistatic sensor working with a constant off-nadir angle, *θ″*;, the parameters of the pendulum formation for four different bistatic off-nadir angles are listed in Table 2.

It is clear that when the satellites move away from the equator the cross track separation decreases and the bistatic satellite leaves the monostatic range elevation plane. Therefore, without envisaging a change of the monostatic/bistatic spacecraft attitude and/or antenna pointing angles, the bistatic coverage is lost. In [35] the following solution is proposed:

- monostatic SAR elevation steering counteracts cross-track separation;
- bistatic SAR azimuth elevation steering (<4°) avoids along-track separations;
- an *ad hoc* designed yaw-steering manoeuvre for the bistatic satellite can be used to overcome swaths relative rotations.

**Figure 6.**
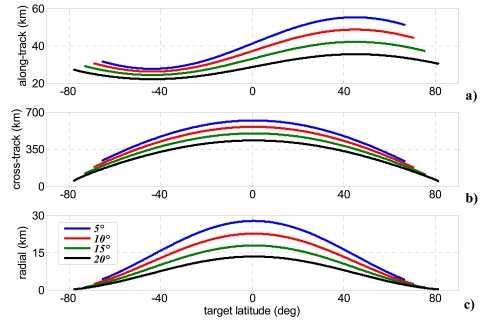
(a) Along-track, (b) cross-track, and (c) radial baseline components for four bistatic antenna off-nadir angles within the range of covered latitudes.

**Figure 7.**
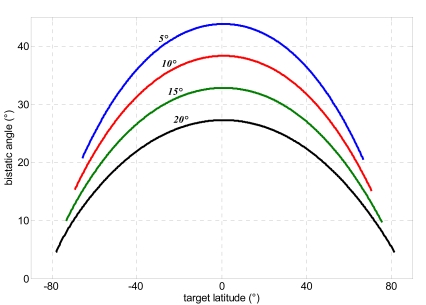
Bistatic angle for four bistatic antenna off-nadir angles within the range of covered latitudes.

**Figure 8.**
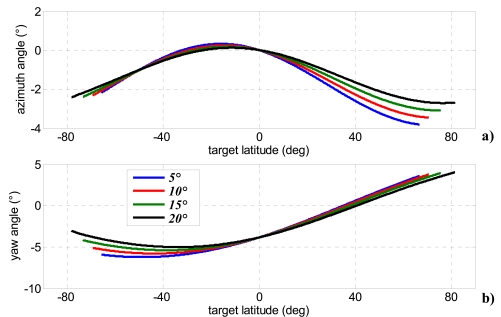
Bistatic antenna azimuth angle (a) and bistatic spacecraft yaw steering angle (b) for four bistatic antenna off-nadir angles within the range of covered latitudes.

This strategy allows bistatic acquisition only during ascending phase within a range of latitudes dependent on the assumed off-nadir angle of the bistatic satellite (see Table 2). It is worth noting that azimuth beam steering can attained in an easier way than elevation steering, since modern SAR antennae are segmented in more tiles in along-track direction, each of them fed separately [3]. In particular, recent studies have pointed out potential, peculiar applications achievable by splitting antenna aperture in along-track direction, such as location of moving targets in high resolution SAR images [36, 37]. Figures 6-8 show baseline components, bistatic angle, and bistatic antenna azimuth and yaw steering angles within the covered latitudes for each of the four selected bistatic off-nadir angles.

## 4. Error Budget of Height Estimation Accuracy

A general approach to the development of an accuracy error budget of each height estimation procedure is to perform a propagation of the height reconstruction error. If only random errors are considered, i.e. assuming that a limited number of ground control points can be used to reduce systematic errors, for any given model:

(15) $$h=h\left(S_{1},S_{2},\ldots ,S_{n}\right)$$

the basic propagation formula for the variance of *h* in terms of all the related error sources *S_1_, S_2,_* …, *S_n_* is:

(16) $$\sigma_{h\;\text{tot}}^{2}=\sum_{i}{\left(\frac{\partial h}{\partial S_{i}}\right)}^{2}\sigma_{S_{i}}^{2}+\sum_{i,j}^{i\neq j}\left|\frac{\partial h}{\partial S_{i}}\frac{\partial h}{\partial S_{j}}\right|\sigma_{S_{i},S_{j}}$$

Table 3 resumes the functional models connected to the methods for monostatic-bistatic DEM generation reported in section 2.2. In a monostatic-bistatic approach, all the parameters can be considered uncorrelated with the exception of the monostatic and bistatic slant ranges [17], therefore:

(17) $$\sigma_{S_{i},S_{j}}\neq 0\;\Leftrightarrow \;\left(S_{i}=r^{\prime },S_{j}=r^{\prime \prime }\right)\;\text{or}\;\left(S_{i}=r^{\prime \prime },S_{j}=r^{\prime }\right)$$

### 4.1. Error Sources and Sensitivities

In order to produce an estimate for height reconstruction error, sensitivities (i.e. the partial derivatives) must be computed and the uncertainty of the error sources must be estimated. Sensitivities can be derived by numerical differentiation of (15) for each method and each orbital position. In more details projection method and hybrid one lead to an explicit formulation for the target altitude that can be differentiated, whereas a least square solution of the bistatic SAR rigorous stereo problem is required to numerically evaluate the sensitivities.

The uncertainties of the monostatic antenna position and velocity depend on the accuracy of the absolute navigation system. A value of 1 m for each component of position and 1 cm/s for each velocity component is compatible with medium accuracy Global Positioning System (GPS) measurements [38, 39]. As regard the baseline and the relative velocity, differential GPS algorithms allow dynamic relative state with decimetre-to-centimetre accuracy to be computed, therefore a conservative value of 0.5 m can be assumed for the uncertainties of each baseline component along with 1cm/s for relative velocity.

Monostatic slant range uncertainties are linked to the dimension, Δ*r′*, of monostatic slant range resolution element [15]:

(18) $$\sigma_{r^{\prime }}^{2}=\frac{1}{12}{\left(\Delta r^{\prime }\right)}^{2}$$

COSMO-SkyMed narrow angle stripmap products are characterized by a final ground range resolution of 3-15 m [40], so values of 3 m and 0.75 m can be assumed, respectively, for ground range and azimuth resolution of single-look-complex data.

Bistatic slant range uncertainty depends on bistatic slant range resolution, as the monostatic one, and it is also related to the error resulting from the application of co-registration procedures required to form stereopairs. A value of 1/10 of an image pixel is compatible with areas where the correlation is high [17]. Hence, bistatic slant range uncertainty can be calculated as follows:

(19) $$\sigma_{r^{\prime \prime }}^{2}=\frac{1}{12}{\left(\Delta r^{\prime }\right)}^{2}+{\left(\frac{\Delta r^{\prime \prime }}{10}\right)}^{2}+{\left(\frac{\Delta x^{\prime \prime }}{10}\right)}^{2}$$

where Δ*r′* is the dimension of bistatic slant range resolution element.

Due to orbital constrains the simple geometry reported in Section 2 must be generalized to tracks that are not perfectly parallel, also accounting for the presence of a time-varying along-track separation between the monostatic and bistatic antennae [Figure 6(a)]. In such conditions (1) and (2) are no longer valid, and more general approaches must be used to calculate bistatic resolutions. The gradient method [26] is used herein, therefore the following resolutions can be obtained:

(20) $$\Delta r^{\prime \prime }=\frac{1}{W}\cdot \frac{1}{\left|\vec{\nabla }t^{\prime \prime }\cdot {\vec{e}}_{r^{\prime \prime }}\right|}$$

(21) $$\Delta {r^{\prime \prime }}_{g}=\frac{1}{W}\cdot \frac{1}{\left|\vec{\nabla }t^{\prime \prime }\cdot {\vec{e}}_{g^{\prime \prime }}\right|}$$

(22) $$\Delta x^{\prime \prime }=\frac{1}{\tau_{\text{int}}}\cdot \frac{1}{\left|\vec{\nabla }{f^{\prime \prime }}_{DC}\cdot {\vec{e}}_{x^{\prime \prime }}\right|}$$

where the bistatic transit time *t′* is:

(23) $$t^{\prime \prime }=\frac{1}{c}\left(r^{\prime }+r^{\prime \prime }\right)$$

and *W* is the receiver bandwidth, ${\vec{e}}_{r^{\prime \prime }}$ is bistatic slant range unit vector, ${\vec{e}}_{g^{\prime \prime }}$ is bistatic ground range unit vector, ${\vec{e}}_{x^{\prime \prime }}$ is bistatic azimuth unit vector, and *τ_int_* is the integration time.

Finally, parallax vector uncertainty, as introduced in section 2.2.3, depends both on the uncertainties of monostatic and bistatic image coordinates and on image co-registration errors. An analytic expression for the contribution of parallax errors to height accuracy is reported in [24]:

(24) $$\sigma_{h}^{2}{|}_{a}=\frac{{{\vec{u}}_{z}}^{T}K_{xy}{\vec{u}}_{z}}{{\left|{\vec{u}}_{z}\right|}^{4}}$$

where:

(25) $$K_{xy}={\vec{e}}_{g^{\prime }}{\vec{e}}_{g^{\prime }}^{T}\sigma_{a_{g}}^{2}+{\vec{e}}_{x^{\prime }}{\vec{e}}_{x^{\prime }}^{T}\sigma_{a_{x}}^{2}$$

(26) $$\sigma_{a_{g}}^{2}=\frac{1}{12}\Delta {r^{\prime }}_{g}^{2}+\frac{1}{12}\Delta {r^{\prime \prime }}_{g}^{2}+{\left(\frac{\Delta {r^{\prime \prime }}_{g}}{10}\right)}^{2}$$

(27) $$\sigma_{a_{x}}^{2}=\frac{1}{12}{\left(\Delta x^{\prime }\right)}^{2}+\frac{1}{12}{\left(\Delta x^{\prime \prime }\right)}^{2}+{\left(\frac{\Delta x^{\prime \prime }}{10}\right)}^{2}$$

${\vec{e}}_{g^{\prime }}$ and ${\vec{e}}_{x^{\prime }}$ are the monostatic ground range and azimuth unit vectors, respectively, and 1/10 of an image pixel is assumed for the accuracy of co-registration procedure again.

### 4.2. Height Estimation Accuracy

**Figure 9.**
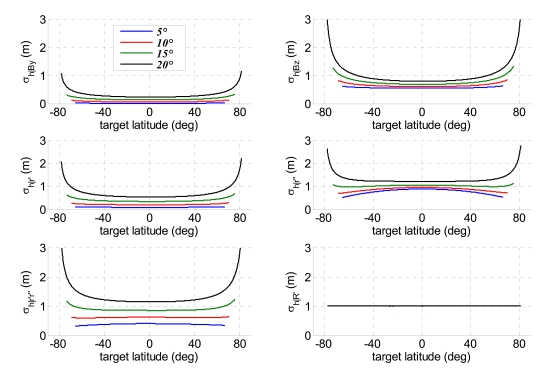
Contributions to height uncertainty as a function of target latitude in the method of projection of bistatic parameters for four bistatic antenna off-nadir angles (1/10 of an image pixel assumed as co-registration uncertainty).

**Figure 10.**
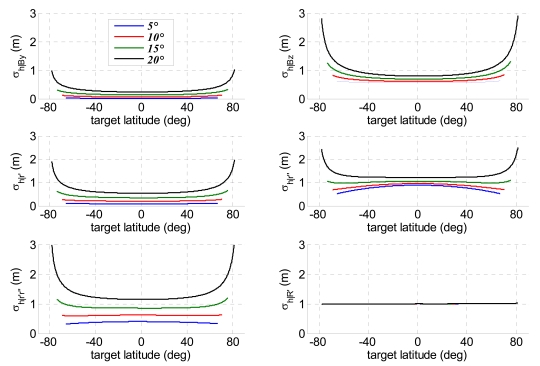
Contributions to height uncertainty as a function of target latitude in the bistatic rigorous SAR stereo problem for four bistatic antenna off-nadir angles (1/10 of an image pixel assumed as co-registration uncertainty).

**Figure 11.**
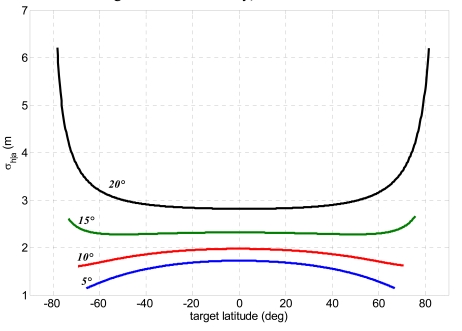
Height uncertainty as a function of target latitude due to vector parallax difference uncertainty in the hybrid method for four bistatic off-nadir angles (1/10 of an image pixel assumed as co-registration uncertainty).

The analysis of the error sources allows evaluation of single contributions to height accuracy for each method. As expected, these contributions are not constant along the orbit since all the sensitivities depend on the actual acquisition geometry, but also the uncertainties related to image resolutions are affected by the instantaneous orbital configuration. Figures 9 and 10 report the principal parameters contributing to relief reconstruction errors, respectively, for projection method and bistatic rigorous SAR stereo problem: *σ_h_*_|_*_By_* and*σ_h_*_|_*_Bz_* are the height uncertainties due to horizontal and vertical baseline components, *B_y_* and *B_z_*; *σ_h_*_|_*_r′_* and*σ_h_*_|_*_r″_* depend on monostatic and bistatic slant range uncertainties, whereas *σ_h_*_|_*_r′r″_* is the height uncertainty relevant to correlated errors in monostatic and bistatic slant ranges; finally, *σ_h_*_|_*_R′_* is the height uncertainty due to monostatic platform positioning errors. Projection method and bistatic rigorous SAR stereo problem share the main error sources and provide very similar results. The largest height uncertainties are due to the vertical baseline components, bistatic slant range accuracy and correlation terms between monostatic and bistatic slant ranges. The contributions of both horizontal baseline components, and monostatic slant range respect the principle of the stereo effect perfectly. Indeed, the larger is the bistatic angle, the lower is the height estimation error. This effect is more manifest when the off-nadir angle of the bistatic sensor is large, while it fades away decreasing *θ″*. Height uncertainties due to bistatic slant range and to the correlations terms deserve more attention: actually the worsening of bistatic slant range resolution with the bistatic angle exceeds the benefits of a better stereo geometry, thus making acceptable the increased height uncertainty for small off-nadir angles of the bistatic sensor [17]. Finally, it is worth noting that an error in monostatic platform position merely produces an identical error on target altitude, a result shared with SAR interferometry [7]. Figure 11 shows the height error due to parallax uncertainty for the hybrid method, representing the single factor causing a meaningful relief reconstruction error. Since the hybrid method is based on the calculation of the parallax from monostatic and bistatic measurements of ground range and azimuth positions [17], it is more sensitive to variation of the bistatic slant range resolution with the bistatic angle.

It is worth noting that previous simulation results show practically constant performances in measurement accuracy within the range of latitudes where stereo radargrammetric coverage can be achieved with satisfactory baselines and bistatic angles, thus demonstrating that the unavoidable non perfectly parallel trajectories and limited along-track separations are not destructive for the quality of height determination.

**Figure 12.**
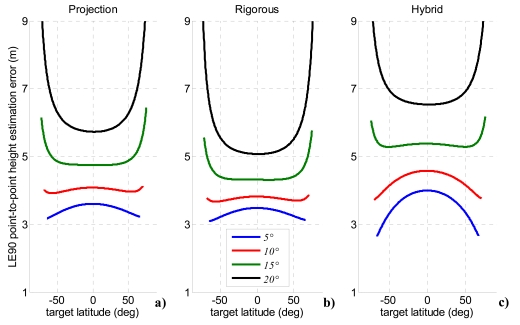
Performance comparison between the analyzed methods for spaceborne bistatic SAR stereo radargrammetry considering 1/10 of an image pixel as co-registration uncertainty and four bistatic off-nadir angles: (a) projection of bistatic parameters, (b) bistatic rigorous stereo SAR problem, (c) hybrid method.

According to international standards assessed to specify DEM accuracy [41], Figure 12 shows the overall relative height uncertainty for each method expressed as point-to-point 90% linear error (LE_90_). The term “point-to-point” indicates the difference of height estimation error between two pixel of the imaged area, while LE_90_ is the error range which include 90% of the pixels.

The height uncertainty exhibits the same order of magnitude for all methods. The best results are obtained when the bistatic off-nadir angle attains lower values (5-10°). This can be explained considering the variation of the bistatic angle for each of the four selected values of the off-nadir angle (Figure 7). Indeed, when the bistatic antenna off-nadir angle is minimum, a larger bistatic angle is established, producing a better stereo effect and thus improving the height estimation accuracy. The small variations of the height uncertainty within the observed latitude can be used to design an orbit (i.e. to select adequate RAAN and anomaly separations) which allows more favourable bistatic angles to be achieved at latitudes where more accurate DEM generation is required.

**Figure 13.**
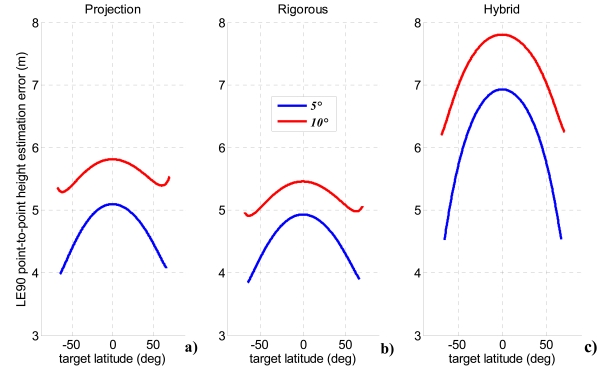
Performance comparison between the analyzed methods for spaceborne bistatic SAR stereo radargrammetry considering 1/2 of an image pixel as co-registration uncertainty and low bistatic off-nadir angles: (a) projection of bistatic parameters, (b) bistatic rigorous stereo SAR problem, (c) hybrid method.

However, it must be noted that the best geometric conditions (*θ′* = 5-10°) could introduce radiometric disparities between monostatic and bistatic images which could influence co-registration algorithms performance [13], hence it is more conservative to consider a larger co-registration error (Figure 13). The method of projection of bistatic parameters and the bistatic rigorous SAR stereo problem are more robust with respect to radiometric disparities leading to a LE90 point-to-point error of 4-5 m over the whole range of covered latitudes, also considering a co-registration error dropped to 1/2 of an image pixel.

**Figure 14.**
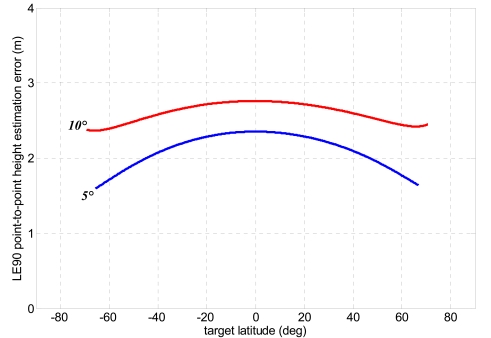
Height estimation error (LE90 point-to-point) as a function of target latitude for bistatic rigorous stereo SAR problem and low bistatic off-nadir angles (1/10 of an image pixel assumed as co-registration uncertainty, 0.25 m as uncertainty for monostatic antenna position error, and 0.1 m as baseline component measurement uncertainty).

Finally, it is possible to provide some guidelines to improve performance. It is clear that a reduction of monostatic resolutions would be useful, however if the reference monostatic mission is held, and present trend is obviously towards high resolution missions, the simplest way to reduce height uncertainty is to upgrade the absolute and relative navigation accuracy. This can be carried out without hardware modifications, but by means of a better filtering of GPS observables. Indeed, in recent years robust algorithms have been developed, able to estimate the absolute position with a decimetre accuracy [42] and the relative position with a centimetre one, even for baselines of hundreds of kilometres [43, 44] and highly variable along the orbit [45]. In this context it is useful to investigate the gain in relief reconstruction accuracy achievable with 25 cm errors on each component of the absolute position and with 10 cm error for the relative baseline, along with 1/10 of an image pixel as co-registration error, representing the best achievable performance on area characterized by favourable conditions of surface coverage and slope characteristics (Figure 14). In this case LE90 point-to-point drops to 1.5–2.5 m.

## 5. Conclusions

The mission design of a spaceborne monostatic-bistatic SAR has been presented in order to evaluate the effects of orbital configurations and pointing strategies on the performance of single-pass stereo-radargrammetric methods for DEM generation. Proposed approach can be fruitfully used to investigate overall constraints and on-orbit configurations of future SAR missions, since it accounts for all main factors affecting measurement accuracy of stereo-radargrammetric applications, such as baseline and antenna state vector knowledge, image quality, monostatic and bistatic resolutions, Doppler parameters, etc. Special emphasis has been given to the application of advanced procedures to derive antenna state vectors based on GPS.

The sensitivity analysis carried out has shown that the so-called parallel orbit configuration offers adequate accuracy in relief reconstruction in spite of the unavoidable non perfectly parallel trajectories and limited along-track separations between monostatic and bistatic antennae due to the relative rotations between the orbital planes and between the satellites within each orbital plane. The effect of the latitude of the observed scene on measurement uncertainty has been pointed out too. Since the proposed model computes quantitatively performance in terrain height evaluation along the orbit, it allows a straightforward selection of the relative positions between the satellites on the ascending node, able to guarantee a higher accuracy over assigned latitude intervals.

Realization in a near future of a dedicated spaceborne monostatic-bistatic SAR mission with large, programmable baselines seems to be compatible with the present generation spaceborne microwave sensors and the current formation flying technologies. Such mission could be able to produce DEM by means of radar-stereogrammetric techniques offering 4-5 m as height accuracy on a global scale within a wide range of surface coverage and slope characteristics. This product could be an indispensable addition of DEMs generated by SAR interferometry both for applications and for engineering goals (as an example to improve phase unwrapping procedures), thanks to the expected robustness of the radargrammetric technique, which is less sensitive to terrain characteristics and decorrelation, and can be fulfilled over larger latitude intervals with almost constant performance, as shown by the investigation.

## Figures and Tables

**Table 1.** Monostatic satellite orbital parameters [18, 19].

Semimajor axis (km)	6,997.9
Inclination (°)	97.87
Eccentricity	0.0018
Argument of perigee (°)	90

**Table 2.** Latitude intervals where satisfactory bistatic baselines and angles are achieved during ascending phase in pendulum configurations for four off-nadir angles of the bistatic sensor.

**Bistatic off-nadir angle**	**RAAN difference**	**Mean anomaly difference**	**Covered latitudes**

5°	5.14°	1.04°	[-65.7°, 66.8°]
10°	4.64°	0.942°	[-69.3°, 70.7°]
15°	4.12°	0.837°	[-73.8°, 75.6°]
20°	3.58°	0.792°	[-78.1°, 81.5°]

**Table 3.** Functional models of proposed monostatic-bistatic methods for terrain relief reconstruction.

**Method**	**Functional Model**

Projection of Bistatic Parameters	$h=h\left(\vec{R},{\vec{V}}_{⊕}^{\prime },\vec{B},r^{\prime },r^{\prime \prime }\right)$
Bistatic rigorous SAR stereo problem	$h=h\left({\vec{R}}^{\prime },{\vec{V}}_{⊕}^{\prime },\vec{B},{\vec{V}}_{⊕}^{\prime },r^{\prime },r^{\prime \prime },f_{DC}^{\prime },f_{DC}^{\prime \prime }\right)$
Hybrid	$h=h\left(\vec{a},{\vec{R}}^{\prime },{\vec{V}}_{⊕}^{\prime },\vec{B},{\vec{V}}_{B⊕}^{\prime }\right)$
